# Transcriptome analysis of ectopic chloroplast development in green curd cauliflower (*Brassica oleracea *L. var. *botrytis*)

**DOI:** 10.1186/1471-2229-11-169

**Published:** 2011-11-23

**Authors:** Xiangjun Zhou, Zhangjun Fei, Theodore W Thannhauser, Li Li

**Affiliations:** 1 Robert W Holley Center for Agriculture and Health, USDA-ARS, Cornell University, Ithaca, NY 14853, USA; 2 Department of Plant Breeding and Genetics, Cornell University, Ithaca, NY 14853, USA; 3 Boyce Thompson Institute for Plant Research, Cornell University, Ithaca, NY 14853, USA

## Abstract

**Background:**

Chloroplasts are the green plastids where photosynthesis takes place. The biogenesis of chloroplasts requires the coordinate expression of both nuclear and chloroplast genes and is regulated by developmental and environmental signals. Despite extensive studies of this process, the genetic basis and the regulatory control of chloroplast biogenesis and development remain to be elucidated.

**Results:**

Green cauliflower mutant causes ectopic development of chloroplasts in the curd tissue of the plant, turning the otherwise white curd green. To investigate the transcriptional control of chloroplast development, we compared gene expression between green and white curds using the RNA-seq approach. Deep sequencing produced over 15 million reads with lengths of 86 base pairs from each cDNA library. A total of 7,155 genes were found to exhibit at least 3-fold changes in expression between green and white curds. These included light-regulated genes, genes encoding chloroplast constituents, and genes involved in chlorophyll biosynthesis. Moreover, we discovered that the cauliflower *ELONGATED HYPOCOTYL5 *(*BoHY5*) was expressed higher in green curds than white curds and that 2616 HY5-targeted genes, including 1600 up-regulated genes and 1016 down-regulated genes, were differently expressed in green in comparison to white curd tissue. All these 1600 up-regulated genes were HY5-targeted genes in the light.

**Conclusions:**

The genome-wide profiling of gene expression by RNA-seq in green curds led to the identification of large numbers of genes associated with chloroplast development, and suggested the role of regulatory genes in the high hierarchy of light signaling pathways in mediating the ectopic chloroplast development in the green curd cauliflower mutant.

## Background

Chloroplast biogenesis from proplastids requires coordinate expression of nuclear and chloroplast genes [1], and is largely regulated by developmental and environmental cues such as light. Approximately 3000 proteins in chloroplasts are encoded by the nucleus [2]. They participate in a large number of functional processes that are required for chloroplast biogenesis. These processes include import of nuclear encoded proteins through the Toc/Tic complexes, protein assembly and disassembly with chaperone proteins, thylakoid formation, pigment synthesis, plastid divisions, and retrograde signaling [3,4]. In addition, a great number of proteins localized outside chloroplasts, such as photoreceptors, light-signaling transducers, and transcription factors, have been shown to be involved in chloroplast development [3,4]. On the one hand, most genes belonging to these two classes are essential for chloroplast development since suppression of their expressions leads to impaired chloroplasts. On the other hand, some light signaling pathway genes, such as *constitutive photomorphogenic 1 *(*COP1*), *COP10*, *COP11*, *De-etiolated 1 *(*DET1*) and *Phytochrome-interacting transcription factor 3 *(*PIF3*), function as suppressors of light-regulated gene expression and loss-of-function mutations of these genes result in ectopic chloroplast development [5-7]. In contrast, *Elongated Hypocotyl 5 (HY5) *that acts downstream of multiple families of photoreceptors [8-10] has been genetically characterized as a positive regulator of photomorphogenesis under a broad spectrum of light and affects chloroplast development [4,11]. Overexpression of HY5-ΔN77 has been shown to result in precocious development of chloroplasts in the hypocotyls [12]. Determining how these genes are coordinately expressed during chloroplast development requires a genome-wide examination of gene expression during the transition from non-colored plastids into chloroplasts.

Mutations in model and other plant species are important resources for functional genomics studies. Analyses of some plastid development mutants identify important regulatory genes of plastid development. For example, *ARC6*, the first gene discovered to have a global effect on plastid differentiation in higher plants, was identified from an Arabidopsis mutant *arc6 *[13]. The *Orange *(*Or*) gene that encodes a zinc-finger DnaJ cysteine rich domain containing protein is isolated from the orange curd cauliflower mutant and has been proven to be responsible for the conversion of leucoplasts into chromoplasts [14]. The green curd cauliflower mutant is a spontaneous mutation with an abnormal pattern of chloroplast development in curds. Compared with other mutants in which chloroplast development is impaired, the green curd mutant is unique in turning otherwise non-photosynthetic white tissue into green color with the ectopic development of chloroplasts in the inflorescence meristematic cells. The mutation in the green curd cauliflower could involve the gene(s) sufficient for chloroplast development, although there is possibility that the white curd cauliflower carries a genetic mechanism for the suppression of chloroplast development, which the green curd mutation would suppress.

In the present study, we profiled gene expression in green and white curds on the genome scale using the RNA-seq approach. We assembled 118,000 unigenes with an average length of 406 bp from cDNA libraries of green and white curds and detected 7155 differentially expressed genes with a change in expression of at least 3-fold. Among them are a large number of genes associated with chloroplast development. We also observed that *BoHY5 *was expressed at higher level in green curds than in white curds and that 2616 HY5-targeted genes were expressed differentially. Among these HY5-targeted genes, all the 1600 up-regulated genes were found to be HY5-targeted genes in the light in Arabidopsis, suggesting a role of *BoHY5 *with the ectopic chloroplast development in the green curd cauliflower mutant.

## Results

### Cauliflower mutant with green curds

**Figure 1:**
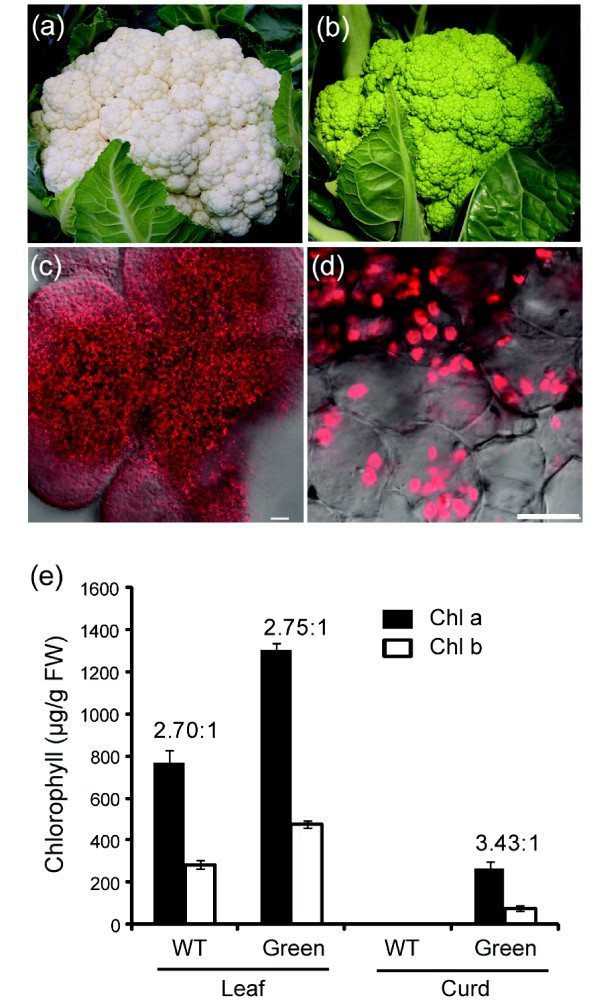
**Phenotype and chlorophyll content of green curd cauliflower mutant**. (a) and (b) Field grown curds from white cauliflower variety (Stovepipe) and green curd line (ACX800), respectively. (c) and (d) Autoflorescence of chloroplasts in green curds. Scale bar in (c) = 20 μm and in (d) = 10 μm. (e) Chlorophyll a and b content in young leaves and curds of Stovepipe (WT) and ACX800 (Green) cauliflower. The numbers above bars show the ratio of chlorophyll a/b. Error bars represent ± SD (n = 3).

Cauliflower curd is composed of inflorescence meristems that normally contain proplastids and leucoplasts and is therefore white [15]. In the commercially available green cauliflower mutant, chloroplasts are developed in the curd, turning the otherwise white tissue green (Figure 1a and 1b). While the mutant plants produced green curds under normal growth conditions in greenhouse and in field, the intensity of green hue in the curd tissues was affected by light intensity. Under field growth conditions, the curd tissues exposed to direct sunlight showed dark green color and those grown in shade exhibited less green hue. Autofluorescence of chlorophyll in chloroplasts was clearly observed in the green curd cells under the confocal microscope (Figure 1c and 1d).

To investigate chloroplast development in the green curd mutant, we first measured chlorophyll content in young leaf and curd tissues. Higher level of total chlorophyll was detected in leaf tissue of green cauliflower plants than that of the white control. The concentration of chlorophyll in green curd cauliflower leaves was 1780.4 μg/g fresh weights (FW), while that in the white curd leaves was 1056.6 μg/g FW. Although different levels of total chlorophyll were observed between the two samples, the ratio of chlorophyll a/b for leaves in white and green mutant was similar at 2.70:1 and 2.75:1, respectively. In comparison to leaf tissue, the chlorophyll level in the curd of green cauliflower was lower at 344.4 μg/g FW. The chlorophyll a/b ratio was 3.43:1, showing that the accumulation of chlorophyll a was much greater than that of chlorophyll b in green curds (Figure 1e). As expected, no chlorophyll accumulation was detected in the white curd tissue. The green curd cauliflower mutant serves as an excellent model system for investigating the genetic basis of chloroplast biogenesis in plants.

### Comparative analysis of gene expression between green and white curd cauliflower

**Figure 2:**
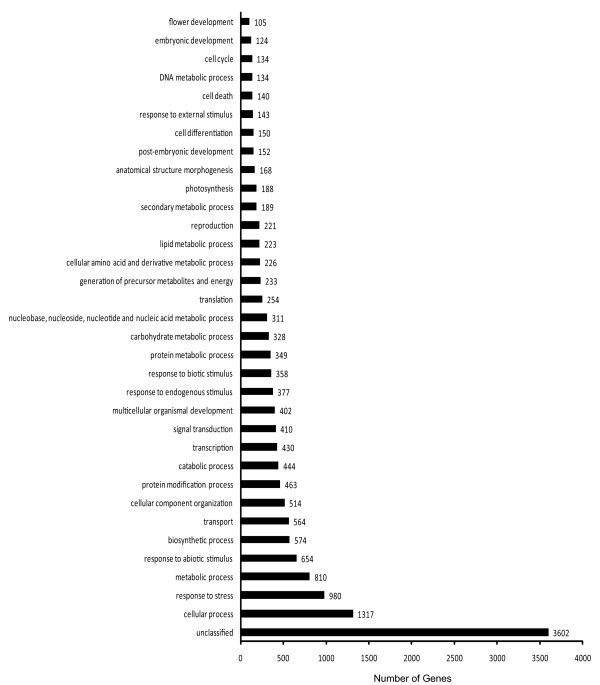
**Functional categories of genes differentially expressed between green and white curds**.

To investigate the transcriptional control of chloroplast development, RNA-seq was employed to monitor differences in gene expression between the green curd mutant and the white cauliflower. A single lane of an Illumina GAII run was utilized for each library and a total of more than 15 million 86-bp reads from each lane were produced. Since currently there is no full genome sequence available for cauliflower (*Brassica oleracea) *and the genomics resources from other *Brassica *species are not applicable due to the short length of RNA-seq reads, we developed a novel analysis strategy for our RNA-seq data as described in the Methods section. A total of 118,000 unigenes (including alternative spliced isoforms) with an average length of 406 bp were obtained. Statistical analysis identified 7155 unigenes that were differentially expressed between green curd mutant and white curd control. Among them, 4436 genes (3.76%) were expressed at least 3-fold higher (Additional file 1) and 2719 genes (2.3%) were expressed at least 3-fold lower in green curd than in white curd (Additional file 2). Functional categorization revealed that these genes were largely involved in cellular process (1317), response to stress (980), metabolic process (810), response to abiotic stimulus (654), and biosynthetic process (574). Yet, a large group of genes (3602) remained unclassified (Figure 2).

### Verification of gene expression by quantitative RT-PCR

In order to verify the expression profiles obtained from the RNA-seq approach, qRT-PCR was utilized to analyze the expression of 14 selected genes. These genes encode light signal transducers (FAR1, CRY2, PHOT2, LSH7, HY5, CIP1), photosystem II component (LHCB5), chloroplast constituents (GUN5, LHCB1.5, Toc159, HSC70-1, ACP), ATP-dependent peptidase (FtsH8) and chlorophyll synthetase (G4). Among them were 11 up-regulated genes (FAR1, CRY2, PHOT2, HY5, G4, Toc159, LHCB5, GUN5, LHCB1.5, FtsH8, and ACP) and 3 down-regulated genes (HSC70-1, CIP1, LSH7). The trends of the observed expression patterns of these genes from qRT-PCR were consistent with that determined by the RNA-seq approach (Table 1). However, there were differences at the fold level as reported in other studies [16].

**Table 1:** Verification of gene expression by qRT-PCR

Genes	RPKM white	RPKM green	Ratio green/white	qRT-PCR Ratio* green/white
Toc159	0	23.1	23.1	4.52
ACP	0	18.7	18.7	1.47
FtsH8	0	12.8	12.8	1.55
LHCB1.5	50.6	320	6.32	7.84
G4	7.8	45.5	5.83	2.7
FAR1	2	8.2	4.1	1.64
CRY2	4.4	18.6	4.23	6.91
HY5	0	7.1	7.1	4.09
PHOT2	2	6.5	3.25	2.06
LHCB5	24.4	90.1	3.69	4.02
GUN5	3.6	14.5	4.03	4.81
CIP1	14.7	4.1	0.28	0.27
LSH7	9.1	1.8	0.2	0.63
HSP70-1	59.2	0.5	0.01	0.0003

*qRT-PCR was carried out with two biological repeats and three technical trials.

### Metabolic pathway changes

To identify the metabolic pathways that were affected in the green curd mutant, a cauliflower metabolic pathway database was created based on annotation of the assembled cauliflower unigenes. The significantly affected pathways were identified by using the Plant MetGenMAP analysis system http://bioinfo.bti.cornell.edu/cgi-bin/MetGenMAP/home.cgi[17]. A total of 198 specific metabolic pathways were significantly changed in green curd mutant (p < 0.01) (Additional file 3). As expected, many metabolic pathways involved in chloroplast biogenesis and function were significantly altered. These included those associated with chlorophyll biosynthesis, such as chlorophyllide a biosynthesis I, chlorophyll a biosynthesis I, chlorophyll a biosynthesis II, chlorophyll a degradation, and chlorophyll cycle, as well as with carotenoid biosynthesis (Additional file 3). In addition, those pathways associated with photosynthesis, such as oxygenic photosynthesis, Calvin cycle, and photorespiration, and with other metabolic processes that take place in chloroplasts, such as amino acid biosynthesis and starch biosynthesis, were also significantly changed (Additional file 3).

### Genes involved in chloroplast formation

**Figure 3:**
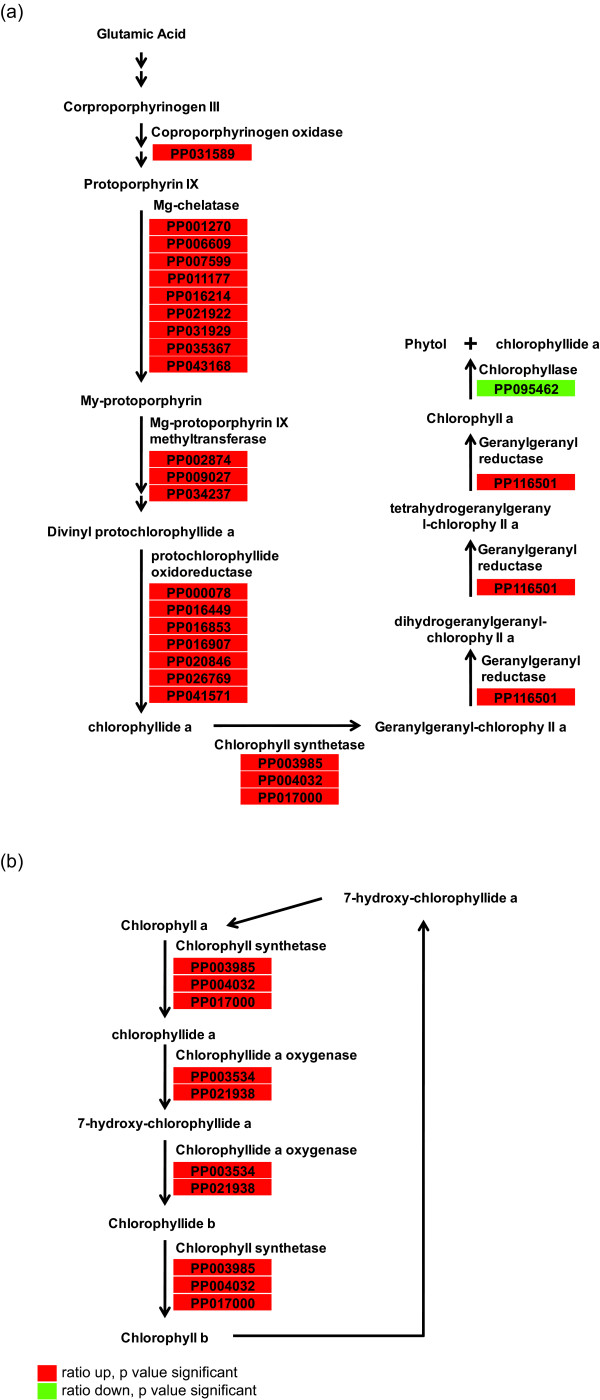
**Alignment of the associated differentially expressed genes with chlorophyll biosynthesis and degradation pathways**. (a) Simplified chlorophyll a biosynthesis and degradation pathway. (b) Chlorophyll cycle. The pathway maps were generated using the Plant MetGenMAP system [17].

Chlorophylls and carotenoids compose the photosynthetic pigments that play key roles in photosynthesis. Many genes involved in chlorophyll biosynthesis were found to be expressed highly in green curd in comparison with white (Figure 3). The upregulated genes included *Mg-chelatase *that plays a key regulatory role in chlorophyll biosynthesis. *Genomes uncoupled 4 *(*GUN4*, PP005347) and *Genomes uncoupled 5 *(*GUN5*, PP031929) involved in chlorophyll biosynthesis were also expressed at higher levels in green curds. These two genes are among those that produce plastid-to-nuclear retrograde signaling molecules [18,19]. The upregulation of many genes in chlorophyll biosynthesis resulted in the accumulation of chlorophyll a and b in chloroplasts. Concomitantly, a number of genes involved in carotenoid biosynthesis were also up-regulated (Table 2), suggesting an increased capacity for the synthesis of photosynthetic pigments. Consistent with the accumulation of chlorophyll a and b in green curds, genes encoding chlorophyll binding proteins were also up-regulated (Table 2). Moreover, genes encoding photosystem I and photosystem II proteins were among the up-regulated genes (Table 2), indicating the development of chloroplast structures in the green curd tissue.

**Table 2:** Genes encoding carotenoid biosynthetic enzymes, chlorophyll binding proteins and photosystem proteins

	RPKM	green/white			Top hit		
unigene ID	white	green	ratio	p value	ID	description*	e value
**Carotenoid biosynthesis**							
PP038070	0.3	7	23.3333	0	AT4G25700.1	CAROTENE BETA-RING HYDROXYLASE	8e-093
PP043130	0.3	4.2	14	0	AT4G25700.1	CAROTENE BETA-RING HYDROXYLASE	6e-036
PP013262	0.7	3.5	5	5.64E-06	AT3G21500.2	1-DEOXY-D-XYLULOSE-5-PHOSPHATE SYNTHASE	1e-032
PP011276	1.1	4.3	3.90909	2.88E-06	AT5G67030.1	ZEAXANTHIN EPOXIDASE	1e-034
PP088927	5.4	18.9	3.5	0	AT4G15560.1	1-DEOXY-D-XYLULOSE-5-PHOSPHATE SYNTHASE	1e-054
							
**Chlorophyll binding**							
PP019996	0.2	36.9	184.5	0	AT1G15820.1	LHCB6	8e-042
PP013404	1.1	100.8	91.6364	0	AT2G34430.1	LHCB1.4	4e-042
PP042072	0.2	12.1	60.5	0	AT1G29930.1	LHCB1.5	4e-047
PP032956	2.5	140.7	56.28	0	AT2G34430.1	LHCB1.4	5e-112
PP036244	0.5	15.5	31	0	AT3G54890.1	LHCA1	1e-131
PP026927	1	26.9	26.9	0	AT3G27690.1	LHCB2.3	4e-059
PP032648	0.5	12	24	0	AT2G34430.1	LHCB1.4	3e-144
PP034454	1.2	25.7	21.4167	0	AT3G27690.1	LHCB2.3	3e-096
PP014055	4.5	90.2	20.0444	0	AT1G29930.1	LHCB1.5	5e-120
PP022096	1.8	34.9	19.3889	0	AT3G54890.1	LHCA1	3e-043
PP016518	5.2	80.2	15.4231	0	AT1G61520.1	LHCA3	1e-096
PP036291	5.6	85.6	15.2857	0	AT3G61470.1	LHCA2	6e-103
PP002791	0.7	10.4	14.8571	0	AT1G15820.1	LHCB6	6e-026
PP060891	0	13.8	13.8	0	AT3G54890.1	LHCA1	3e-037
PP032891	0.7	9.4	13.4286	0	AT5G01530.1	LHCB4.1	2e-039
PP033574	1.4	18.1	12.9286	0	AT3G54890.1	LHCA1	9e-121
PP043522	2.1	25.6	12.1905	0	AT1G61520.1	LHCA3	5e-036
PP004292	6.9	83.9	12.1594	0	AT3G61470.1	LHCA2	4e-132
PP041756	1.1	13.3	12.0909	0	AT2G34430.1	LHCB1.4	6e-023
PP004529	21.5	259.4	12.0651	0	AT1G15820.1	LHCB6	1e-128
PP003367	14.8	176.1	11.8986	0	AT3G61470.1	LHCA2	7e-125
PP019899	28.5	328.4	11.5228	0	AT1G29930.1	LHCB1.5	2e-146
PP020291	40.5	445.8	11.0074	0	AT2G34430.1	LHCB1.4	1e-143
PP055138	0.7	7.6	10.8571	0	AT3G27690.1	LHCB2.3	9e-048
PP005460	2.1	21.5	10.2381	0	AT3G54890.2	LHCA1	9e-066
PP020373	0	9.9	9.9	0	AT3G08940.2	LHCB4.2	3e-025
PP021872	12.4	115	9.27419	0	AT3G08940.2	LHCB4.2	1e-144
PP034445	0.9	8.3	9.22222	0	AT3G27690.1	LHCB2.3	3e-099
PP003971	13.1	117.1	8.93893	0	AT3G54890.1	LHCA1	1e-135
PP034046	1.1	9.2	8.36364	0	AT5G54270.1	LHCB3	4e-153
PP033656	4	31.3	7.825	0	AT5G54270.1	LHCB3	1e-028
PP029126	6	46.1	7.68333	0	AT1G61520.1	LHCA3	3e-077
PP005284	6.7	47.7	7.1194	0	AT1G29930.1	LHCB1.5	2e-105
PP021031	36.8	258.7	7.02989	0	AT2G34430.1	LHCB1.4	1e-061
PP016372	2.4	16.7	6.95833	0	AT2G34430.1	LHCB1.4	7e-020
PP022981	6.9	47	6.81159	0	AT2G34430.1	LHCB1.4	6e-060
PP021682	14.1	91.5	6.48936	0	AT1G61520.1	LHCA3	2e-130
PP003968	50.6	320	6.32411	0	AT1G29930.1	LHCB1.5	9e-151
PP020586	22.7	135.7	5.97797	0	AT1G61520.1	LHCA3	3e-129
PP005425	5.4	26.2	4.85185	0	AT1G29910.1	LHCB1.5	1e-051
PP010147	48.6	226.7	4.66461	0	AT1G15820.1	LHCB6	5e-123
PP003504	66.3	305.1	4.60181	0	AT3G47470.1	LHCA4	8e-131
PP004840	40.6	181.7	4.47537	0	AT2G34430.1	LHCB1.4	4e-115
PP020865	14.6	61.6	4.21918	0	AT2G34430.1	LHCB1.4	2e-019
PP032222	8.6	34.2	3.97674	0	AT4G10340.1	LHCB5	7e-098
PP021166	104.1	391.8	3.76369	0	AT2G34430.1	LHCB1.4	6e-150
PP005062	24.4	90.1	3.69262	0	AT4G10340.1	LHCB5	2e-128
PP033260	4.3	15.8	3.67442	0	AT3G27690.1	LHCB2.3	3e-140
PP032950	102.9	376.3	3.65695	0	AT2G34430.1	LHCB1.4	5e-150
PP022979	10	33	3.3	0	AT1G44575.1	NPQ4	6e-068
PP001298	3.7	12	3.24324	0	AT5G01530.1	LHCB4.1	3e-031
PP021716	24.4	78.8	3.22951	0	AT4G10340.1	LHCB5	1e-133
PP003549	24.1	74.3	3.08299	0	AT1G44575.1	NPQ4	1e-107
							
**Photosystem proteins**							
PP008642	0.2	25.8	129	0	AT3G21055.1	PSBTN (PHOTOSYSTEM II SUBUNIT T)	5e-027
PP000591	0.5	54.9	109.8	0	AT1G03130.1	PSAD-2 (PHOTOSYSTEM I SUBUNIT D-2)	1e-053
PP006879	0.1	4.7	47	0	P11594	OXYGEN-EVOLVING ENHANCER PROTEIN 2	2e-012
PP004388	1.1	45.3	41.1818	0	AT1G55670.1	PSAG (PHOTOSYSTEM I SUBUNIT G)	1e-066
PP016988	0.2	4.9	24.5	0	AT2G30570.1	PSBW (PHOTOSYSTEM II REACTION CENTER W)	5e-025
PP018045	7.8	174.6	22.3846	0	AT1G08380.1	PSAO (PHOTOSYSTEM I SUBUNIT O)	8e-070
PP036218	0.3	5	16.6667	0	ATCG00280.1	CP43 SUBUNIT OF THE PHOTOSYSTEM II REACTION CENTER	2.00E-171
PP032734	1.1	17.4	15.8182	0	AT1G08380.1	PSAO (PHOTOSYSTEM I SUBUNIT O)	1e-065
PP020420	14.5	221.7	15.2897	0	AT1G06680.1	PSBP-1 (PHOTOSYSTEM II SUBUNIT P-1)	9e-122
PP018042	0	14.7	14.7	0	AT1G52230.1	PSAH2 (PHOTOSYSTEM I SUBUNIT H2)	2e-019
PP004848	3.4	49.6	14.5882	0	AT1G06680.1	PSBP-1 (PHOTOSYSTEM II SUBUNIT P-1)	3e-118
PP014192	7.3	103.1	14.1233	0	AT3G21055.1	PSBTN (PHOTOSYSTEM II SUBUNIT T)	6e-036
PP021663	4.9	63.5	12.9592	0	AT2G06520.1	PSBX (PHOTOSYSTEM II SUBUNIT X)	1e-038
PP069357	0.7	8.2	11.7143	0	AT3G21055.1	PSBTN (PHOTOSYSTEM II SUBUNIT T)	1e-035
PP005143	6.3	68.6	10.8889	0	AT4G12800.1	PSAL (PHOTOSYSTEM I SUBUNIT L)	5e-096
PP016409	7.1	60.7	8.5493	0	AT1G08380.1	PSAO (PHOTOSYSTEM I SUBUNIT O)	2e-065
PP033894	5.6	44.7	7.98214	0	AT1G03600.1	PHOTOSYSTEM II FAMILY PROTEIN	2e-051
PP014928	10.7	84	7.85047	0	AT1G30380.1	PSAK (PHOTOSYSTEM I SUBUNIT K)	5e-057
PP017397	10.6	76.7	7.23585	0	AT2G06520.1	PSBX (PHOTOSYSTEM II SUBUNIT X)	2e-018
PP060944	0	7.1	7.1	0	AT1G52230.1	PSAH2 (PHOTOSYSTEM I SUBUNIT H2)	4e-016
PP017005	0	6.6	6.6	0	AT1G79040.1	PSBR (PHOTOSYSTEM II SUBUNIT R)	2e-034
PP021626	0	6.5	6.5	0	AT1G52230.1	PSAH2 (PHOTOSYSTEM I SUBUNIT H2)	1e-035
PP000042	7.8	48.3	6.19231	0	AT1G52230.1	PSAH2 (PHOTOSYSTEM I SUBUNIT H2)	2e-056
PP022192	25.2	130.2	5.16667	0	AT3G50820.1	PSBO2 (PHOTOSYSTEM II SUBUNIT O-2)	4e-176
PP032949	6.7	33.7	5.02985	0	AT1G79040.1	PSBR (PHOTOSYSTEM II SUBUNIT R)	2e-060
PP032493	5.2	25.1	4.82692	0	AT1G52230.1	PSAH2 (PHOTOSYSTEM I SUBUNIT H2)	2e-056
PP033323	21.5	100.9	4.69302	0	AT1G79040.1	PSBR (PHOTOSYSTEM II SUBUNIT R)	4e-061
PP033241	6	27.1	4.51667	0	AT4G03280.1	PETC (PHOTOSYNTHETIC ELECTRON TRANSFER C)	3e-107
PP033302	7.4	32.5	4.39189	0	AT1G79040.1	PSBR (PHOTOSYSTEM II SUBUNIT R)	3e-064
PP005186	48.6	205.7	4.23251	0	AT1G06680.1	PSBP-1 (PHOTOSYSTEM II SUBUNIT P-1)	7e-116
PP016053	22.9	96.6	4.21834	0	AT2G30570.1	PSBW (PHOTOSYSTEM II REACTION CENTER W)	3e-042
PP022547	5.8	23.8	4.10345	0	AT2G06520.1	PSBX (PHOTOSYSTEM II SUBUNIT X)	3e-018
PP012172	7.3	29.7	4.06849	0	AT1G03600.1	PHOTOSYSTEM II FAMILY PROTEIN	6e-066
PP000003	17	68.8	4.04706	0	AT1G55670.1	PSAG (PHOTOSYSTEM I SUBUNIT G)	8e-067
PP033216	12.2	47.9	3.92623	0	AT1G79040.1	PSBR (PHOTOSYSTEM II SUBUNIT R)	2e-054
PP041363	2	7.7	3.85	0	NP_174418	PSAF (PHOTOSYSTEM I SUBUNIT F)	4e-009
PP012010	26.9	95.3	3.54275	0	AT4G12800.1	PSAL (PHOTOSYSTEM I SUBUNIT L)	5e-101
PP033278	11.6	40.2	3.46552	0	AT1G79040.1	PSBR (PHOTOSYSTEM II SUBUNIT R)	6e-044
PP033227	10.1	33.6	3.32673	0	AT1G52220.1	PSI-P (PHOTOSYSTEM I P SUBUNIT)	4e-042
PP005055	24	76.9	3.20417	0	AT1G30380.1	PSAK (PHOTOSYSTEM I SUBUNIT K)	3e-055
PP039593	2.1	6.5	3.09524	0	AT1G31330.1	PSAF (PHOTOSYSTEM I SUBUNIT F)	9e-036
PP013176	7	21.5	3.07143	0	AT2G46820.1	PSI-P (PHOTOSYSTEM I P SUBUNIT)	2e-044

*That the top hits of several cauliflower unigenes correspond to one gene in Arabidopsis could be due to paralogs or alternative splicing of genes in cauliflower.

In addition to the enhanced biosynthesis of photosynthetic apparatus, genes involved in a number of other chloroplast biogenesis processes were also differentially expressed in green curd mutant. TRANSLOCON AT THE OUTER ENVELOPE MEMBRANE OF CHLOROPLASTS 34 (Toc34) and Toc159 are important parts of the Toc/Tic complexes mediating protein import from cytosol [1]. High levels of *Toc34 *(PP019500 and PP051864) and *Toc159 *(PP013646 and PP007289) transcripts were observed in the green curds. Proteins imported into chloroplasts need to be properly assembled and folded, a process that is mediated by a group of chaperone proteins, such as HSP70 and Cpn60, and protein disulfide isomerase [3,20,21]. Accordingly, chaperone HSP70 (PP031462, PP020739, and PP094292) and protein disulfide isomerase (PP012584, PP000051, and PP028760) were found to be significantly upregulated in green curds (Additional file 1).

Chlorophyllase catalyzes degradation of chlorophyll a to yield chlorophyllide and phytol [22]. *Chlorophyllase *(PP095462) was expressed lower in green curds than in white curds, which could account for the accumulation of chlorophyll a in green curds (Figure 3).

### Signaling genes for chloroplast biogenesis

The large number of differentially expressed genes between the green curd mutant and white curd cauliflower suggests that genes at high hierarchy in the signal transduction cascade could be involved. COP/DET/FUS are a group of evolutionarily conserved proteins that represent central repressors of photomorphogenesis including chloroplast development [11]. No changes were detected in the expression of *COP1*, *COP10*, *COP11*, and *DET1*. COP9 complex acts as a suppressor of chloroplast development [5,23]. Unexpectedly, we found that *COP9 *(PP010178) and *FUSCA 12 *(*FUS12*)/*COP9 signalosome complex subunit 2 *(PP014936) were expressed at higher levels in green curds than in white curds. Such higher expression could be a result of a negative feedback as the case of SPA1, a partner of COP1, which is frequently found to be light induced [24,25]. PIFs are another group of regulators that repress photomorphogenesis. No changes were observed for the expression of *PIF3 *and *PIF4 *in the green vs. white curds. Interestingly, the transcript of *PIL2 *(PP058986) was increased in the green curd mutant.

**Figure 4:**
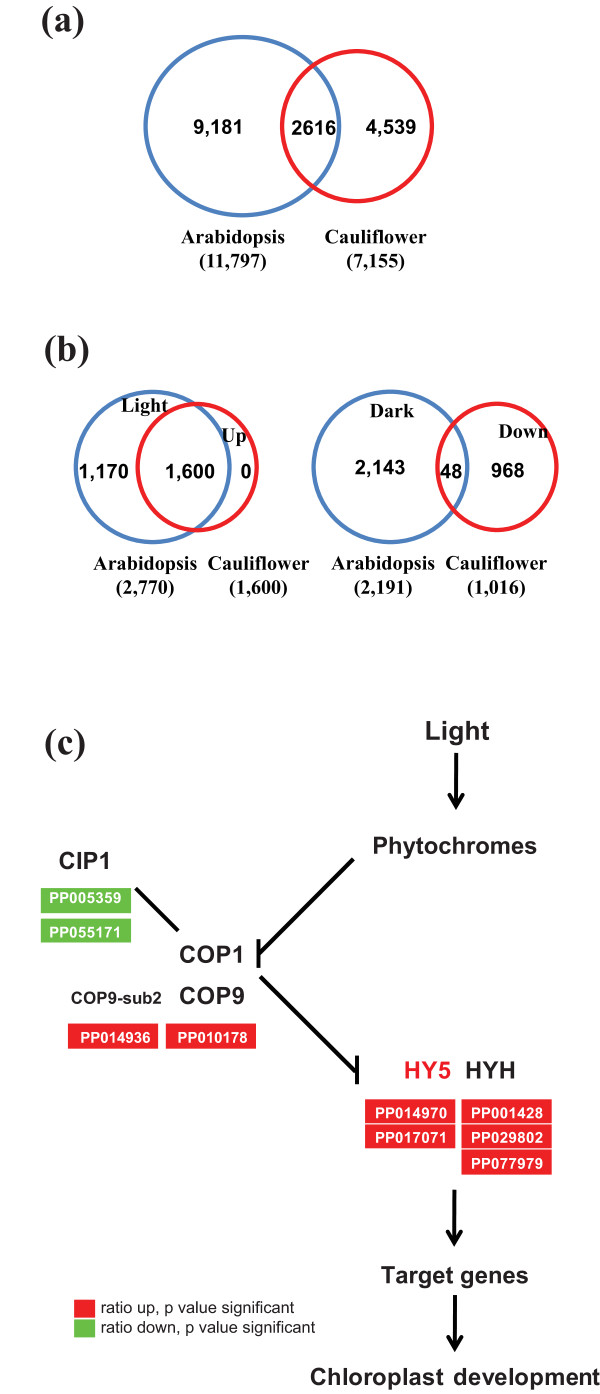
**Comparison of the numbers of *Hy5*-mediated genes in Arabidopsis **[26]**with differentially expressed genes in green curds of cauliflower and a simplified model of light signaling pathway for chloroplast development based on **[11]. (a) Venn diagram showing the number of common genes between HY5-targeted genes in Arabidopsis and total differentially expressed genes in green curds of cauliflower. (b) Venn diagrams showing the numbers of common genes between HY5-regulated genes in Arabidopsis under light and dark and the up- and down-expressed genes among the 2616 homologs of HY5-trageted genes in green curds of cauliflower. (c) Alignment of the associated differentially expressed genes with light signaling pathway for chloroplast development. Abbreviations are as follows: CIP1, COP1-INTERACTIVE PROTEIN 1; COP1, CONSTITUTIVE PHOTOMORPHOGENIC 1; COP9, CONSTITUTIVE PHOTOMORPHOGENIC 9; COP9-sub2, COP9 SIGNALOSOME COMPLEX SUBUNIT 2; HY5, ELONGATED HYPOCOTYL 5; HYH, HY5-HOMOLOG.

In contrast to those photomorphogenesis repressors, HY5 is a key regulator that promotes photomorphogenic development in all light conditions and directly regulates the light-responsive gene expression [8,9,26-28]. Here, we found that *BoHY5 *(PP014970 and PP017071) and *BoHY5-HOMOLOG *(PP001428) were expressed at higher levels in green curds than white curds (Table 1 and Figure 4c). A recent study on genome-wide mapping of the *Hy5*-mediated gene networks in Arabidopsis reveals that HY5 could potentially bind to 11,797 genes with 2770 and 2191 being light and dark regulated genes, respectively [26]. Sequence comparison with the HY5-targeted genes in Arabidopsis revealed that a total of 2616 cauliflower HY5-targeted homolog genes were differentially expressed in green curds (Figure 4a). Among them included 1600 up-regulated genes and 1016 down-regulated genes (Additional file 4 and 5). All of the 1600 up-regulated genes were found to be HY5-targeted genes in the light, while 48 down-regulated genes were HY5-targeted genes in the dark (Figure 4b). Among the 1600 up-regulated HY5-targeted genes were 98 transcription factors, including *ARABIDOPSIS THALIANA HOMEOBOX 1 *(*ATHB-1*, PP003454), *PHYTOCHROME-ASSOCIATED PROTEIN 1 *(PP002569), *PHYTOCHROME INTERACTING FACTOR 3-LIKE 2 *(PP058986) and *INDOLE-3-ACETIC ACID INDUCIBLE *(*IAA1*, PP013005). Forty-four transcription factors including *RAP2.2 *(PP072648), *APETALA1 *(PP029050), *AUXIN RESPONSE FACTOR 6 *(PP006891), and *SHORT HYPOCOTYL 2 *(PP011787) were down-regulated in green curds. The significant alteration of a large number of transcription factors could cause profound effects on chloroplast biogenesis and/or other processes.

## Discussion

Large-scale transcriptome sequencing by next generation sequencing platforms such as the Illumina GA sequencing system has been proven to be a powerful and efficient approach for gene expression analysis at the genome level and offers several advantages over microarray technologies [29]. Since the RNA-seq approach provides digital representation of the gene abundance and the statistics are well modeled by the Poisson distribution, even a single replication has been shown to be adequate [30]. Currently, the RNA-seq approach has been widely used to investigate transcriptomes of plants and animals, especially for those having whole genome sequences [31]. A number of tools to map RNA-seq data to reference genomes and to quantify the expression of transcripts have been developed [32]. However, relatively fewer reports have shown studies on using the RNA-seq approach for organisms without reference genomes. In this report we employed the RNA-seq approach to investigate the gene expression changes in a green curd mutant in order to elucidate the genetic basis of chloroplast biogenesis and development. RNA-seq reads along with publicly available ESTs of cauliflower were assembled *de novo *using a novel assembly strategy as described in the Methods section. A total of 118, 000 unigenes were obtained and 7155 genes showed at least 3-fold changes in expression in green curd mutant. Among them, a large number of genes involved in photomorphogenesis including chloroplast development were revealed, demonstrating a successful use of the RNA-seq approach to profile gene expression in a species without a fully sequenced genome.

Chloroplast biogenesis and development proceed with the coordinated action of many processes [3,4]. Both environmental signals and plastidic/nuclear factors affect these processes. Light regulation of chloroplast development has been well-documented [3,4,33]. The light signaling pathways are composed of phytochromes, transcription factors and numerous intermediates which control photomorphogenesis including chloroplast development. The COP/DET/FUS proteins are suggested to have a function in suppressing chloroplast development in non-photosynthetic tissues [4]. Loss of function mutation of these regulators, such as *cop1 *and *det1*, has been shown to result in ectopic chloroplast development, leading to greening in Arabidopsis roots [5,6]. The fact that the transcripts of *COP1 *and *DET1 *remained unchanged and a large number of light-responsive genes were altered in green curds of cauliflower suggests that other regulatory genes in the hierarchy of photomorphogenic regulation are responsible for chloroplast development in the green curd.

In the light signaling cascade, HY5 plays an important role in light signaling and chloroplast development. HY5 receives upstream signals and activates a large number of genes by directly binding to the G-box in the promoters of these genes [9,26,27]. Here, we observed higher level of *HY5 *transcript in the green curd mutant. Furthermore, 2616 cauliflower homologs of HY5-targeted genes were differentially expressed in green curds. Noticeably, among the 2616 genes, 1600 were up-regulated genes in green curd cauliflower. The fact that all 1600 up-regulated genes were the HY5-targeted genes in the light suggests an important role of elevated expression of *BoHY5 *in mediating chloroplast development in green curd cauliflower mutant. Furthermore, it is known that COP1 negatively controls HY5 activity [12]. Although *COP1 *was expressed at the same level between green curds and white curds, we found that *CIP1 *was significantly reduced in green curds (Figure 4c). Arabidopsis CIP1 is associated with the cytoskeleton and has been hypothesized to affect partitioning of COP1 in the nucleus and cytoplasm [34]. It is possible that COP1 activity in the nucleus might be affected by low level of CIP1, causing ectopic chloroplast development in green curds. Thus, *BoHY5 *and/or the other genes at the high hierarchy in the signal transduction cascade could be responsible or work in concert to regulate chloroplast biogenesis and development in otherwise white tissue to give rise to the striking green curd mutant phenotype.

Ultimately, the development of chloroplasts requires the coordinated action of a number of processes, including the biosynthesis of photosynthetic complexes, transportation of nuclear encoded proteins into chloroplasts, processing of the imported proteins, and assembly of the photosynthetic apparatus [3,4]. Indeed, many genes involved in photosynthetic pigment biosynthesis along with pigment-binding proteins such as chlorophyll a/b binding proteins were discovered to be upregulated in our genome-wide profiling of green curd cauliflower. The majority of chloroplast proteins are nucleus-encoded and enter the chloroplasts via the Toc/Tic translocon complexes [1]. The increased expression of *Toc *genes in the green curd mutant supports an enhanced activity of chloroplast-targeted protein import. The imported proteins are folded and processed to form functional proteins. Molecular chaperones HSP70 and Cpn60 have long been known to be involved in this process [3,20]. A recent study shows that a protein disulfide isomerase is also required for protein folding [21]. Consistent with the increased activity of protein import, genes associated with protein folding and assembling were expressed highly in the green curd mutant for chloroplast development.

## Conclusions

In the present study, we compared gene expression on a genome-wide scale by using RNA-seq in a species without a reference genome. This study identified a great number of genes associated with chloroplast development and suggested the potential role of elevated expression of *BoHY5 *and/or other regulatory genes in the high hierarchy of light signaling pathways for the ectopic chloroplast development in green curd cauliflower. Our results indicate that RNA-seq as a powerful tool in a genomic era could accelerate the functional identification of genes and aid in dissecting the genetic basis of naturally-occurring variations in crops.

## Methods

### Plant materials

White curd cauliflower cultivar Stovepipe (*Brassica oleracea *L. var. *botrytis*) and the green curd mutant line ACX800 were used in this study. Cauliflower plants were grown either in a greenhouse under 14-h-light/10-h-dark cycle at 23°C or in a field. In the greenhouse, the natural daylight was supplied by full-spectrum lamps with the light intensity at 400 μmol photons m^- 2 ^s^-1^. Fresh curd tissues were harvested, immediately frozen in liquid nitrogen, and stored at -80°C for RNA extraction and chlorophyll extraction.

### RNA extraction and construction of cDNA library for sequencing

Total RNA was extracted from pooled curd tissue using the TRIzol reagent according to the manufacturer's instruction (Invitrogen, Carlsbad, CA), and was further purified with the RNeasy^®^Plant Mini Kit (Qiagen, Valencia, CA). The cDNA libraries of green and white cauliflower from five micrograms of total RNA were constructed using the mRNA Sequencing Sample Preparation Kit following the manufacturer's instruction (Illumina, San Diego, CA, USA). Sequencing was carried out on an Illumina/Solexa Genome Analyzer II system at the Cornell University Life Sciences Core Laboratories Center.

### RNA-seq data processing and analysis

The raw Illumina RNA-seq reads were first processed to remove low quality regions and adaptor sequences using an in-house perl script. To eliminate rRNA sequence contamination, the reads were then aligned to cauliflower ribosomal RNA (rRNA) sequences using Bowtie [35], allowing up to two mismatches. A total of ~60,000 cauliflower Sanger ESTs were collected from GenBank in June, 2010. These ESTs were screened against the NCBI UniVec database, the *Escherichia coli *genome, and cauliflower rRNA sequences, to remove those contaminant sequences. The resulting high quality ESTs were assembled into unigenes using iAssembler http://bioinfo.bti.cornell.edu/tool/iAssembler. The processed Illumina reads were then aligned to the cauliflower EST-unigenes using Bowtie [35], allowing up to two mismatches. A *de novo *assembly of the unaligned reads was then performed using ABySS [36]. The unigenes assembled from ESTs and unaligned Illumina reads, respectively, were further assembled using iAssembler. Following mapping to EST-unigenes and *de novo *assembly, transcript count information for sequences corresponding to each unigene were compared to obtain relative expression levels following normalization to RPKM (reads per kilobase of exon model per million mapped reads) [37]. The significance of differential gene expression between the green and white curds was determined using the R statistical method described by Stekel *et al*. [38] and raw p-values were adjusted for multiple tests using the false discovery rate [39]. Genes with a false discovery rate ≤ 0.01 and a fold change no less than 3 were identified as differentially expressed genes between green and white curds.

To identify biological processes affected in the green curd mutant, the differentially expressed genes were annotated by assigning gene ontology (GO) terms. Potential roles of differentially expressed genes in some specific biological processes were identified. In addition, we created a metabolic pathway database based on the annotation information of the assembled cauliflower unigenes using the Pathway Tools [40]. The pathway database was then integrated into the Plant MetGenMAP system [17] to identify the significantly affected pathways.

### Verification of RNA-Seq by quantitative RT-PCR

The cDNA was synthesized using oligo-dT primers and Superscript^® ^reverse transcriptase III (Invitrogen, Carlsbad, CA). qRT-PCR was conducted by using the SYBR Green PCR master mix (Applied Biosystems, CA). The cycling conditions involved denaturation at 95°C for 10 min, followed by 40 cycles of 95°C for 15 s and 60°C for 60 s. The dissociation curves were analyzed to verify the specificity of RT-PCR. The relative expression of selected genes was normalized to a cauliflower actin gene [14]. Values reported represent the average of two biological repeats with three independent trials. Gene-specific primers used are listed in Additional file 6.

### Chlorophyll determination

Fifty milligrams of curds were ground in liquid nitrogen, and 1 mL of 80% acetone was added to extract chlorophyll. After centrifugation at 12,000 g for 5 min, the supernatant was transferred into the new tube and measured at OD_645 _and OD_663_. Chlorophyll concentrations were calculated by using MacKinney's coefficients in the following equations: Chlorophyll a = 12.7*(OD_663_)-2.69*(OD_645_) and Chlorophyll b = 22.9*(OD_645_)-4.48*(OD_663_) [41].

### Confocal analysis of chloroplasts in green curds

Fresh green curd cauliflower tissue was hand-sectioned and examined under Leica TCS SP5 Laser Scanning Confocal Microscope (Leica Microsystems, Exon, PA USA) to detect the autofluorescence of chlorophyll with argon laser excitation at 488 nm and emission filter at 680 nm.

## List of abbreviations

COP1: CONSTITUTIVE PHOTOMORPHOGENIC 1; DET1: DE-ETIOLATED 1; HY5: ELONGATED HYPOCOTYL 5; PHYA: PHYTOCHROME A; PIF3: PHYTOCHROME-INTERACTING TRANSCRIPTION FACTOR 3; FAR1: FAR-RED-IMPAIRED RESPONSE 1; CRY2: CRYPTOCHROME 2; PHOT2: PHOTOTROPIN 2; LSH7: LIGHT SENSITIVE HYPOCOTYLS 7; CIP1: COP1-INTERACTIVE PROTEIN 1; LHCB5: LIGHT HARVESTING COMPLEX OF PHOTOSYSTEM II 5; GUN5: GENOMES UNCOUPLED 5; LHCB1.5: PHOTOSYSTEM II LIGHT HARVESTING COMPLEX GENE 1.5; Toc159: TRANSLOCON AT THE OUTER ENVELOPE MEMBRANE OF CHLOROPLASTS 159; HSC70-1: CHLOROPLAST HEAT SHOCK PROTEIN 70-1; FtsH8: ATP-dependent peptidase/ATPase/metallopeptidase; G4: chlorophyll synthetase.

## Authors' contributions

XZ designed the research, conducted molecular and biochemical analyses, and wrote the manuscript. ZF performed the bioinformatics data analysis. TWT participated in the initial design and discussion of the project, and editing of the manuscript. LL conceived the research and participated in the writing of the manuscript. All authors read and approved the final version of the manuscript.

## Supplementary Material

Additional file 1 **Up-regulated genes in green curd cauliflower mutant**. Click here for file

Additional file 2 **Down-regulated genes in green curd cauliflower mutant**. Click here for file

Additional file 3 **Significantly changed pathways in green curd cauliflower mutant**. Click here for file

Additional file 4 **Up-regulated HY5-targeted genes in green curds**. Click here for file

Additional file 5 **Down-regulated HY5-targeted genes in green curds**. Click here for file

Additional file 6 **Primer sequences used in this study**. Click here for file
